# Exposures, Symptoms and Risk Perception among Office Workers in Relation to Nanoparticles in the Work Environment

**DOI:** 10.3390/ijerph19105789

**Published:** 2022-05-10

**Authors:** Hans Orru, Henrik Olstrup, Annika Hagenbjörk, Steven Nordin, Kati Orru

**Affiliations:** 1 Section of Sustainable Health, Department of Public Health and Clinical Medicine, Faculty of Medicine, Umeå University, 901 87 Umeå, Sweden; annika.hagenbjork@umu.se; 2 Institute of Family Medicine and Public Health, Faculty of Medicine, University of Tartu, Ravila 19, 50411 Tartu, Estonia; 3 Department of Psychology, Faculty of Social Sciences, Umeå University, 901 87 Umeå, Sweden; steven.nordin@umu.se; 4 Institute of Social Studies, University of Tartu, Lossi 36, 51003 Tartu, Estonia; kati.orru@ut.ee

**Keywords:** nanoparticles, exposure, risk perception, work environment, SBS, path analysis

## Abstract

Submicroscopic nanoparticles (NPs) in air have received much attention due to their possible effects on health and wellbeing. Adverse health impacts of air pollution may not only be associated with level of exposure, but also mediated by the perception of the pollution and by beliefs of the exposure being hazardous. The aim of this study was to test a model that describes interrelations between NP pollution, perceived air quality, health risk perception, stress, and sick building syndrome. In the NanoOffice study, the level of NPs was measured and a survey on health risk perception was conducted among 260 employees in twelve office buildings in northern Sweden. Path analyses were performed to test the validity of the model. The data refute the model proposing that the NP exposure level significantly influences stress, chronic diseases, or SBS symptoms. Instead, the perceived exposure influences the perceived risk of NP, and the effect of perceived exposure on SBS and chronic disease is mediated by stress. There was little concern about nanoparticles, despite relatively high levels in some facilities. Perceived pollution and health risk perception may explain a large part of the environmentally induced symptoms and diseases, particularly in relatively low levels of pollution. The research results raise important questions on the physiologically or psychologically mediated health effects of air pollution.

## 1. Introduction

In recent years, concern about airborne nanoparticles (NPs) has increased due to their small size and health risks [1,2,3]. A nanoparticle is defined as a submicroscopic particle that measures less than 100 nanometers (nm) on at least one of its dimensions [4]. Ambient NPs can penetrate indoors from outdoors, and there are also indoor sources [5,6]. The significant health effects may stem from the fact that the particles of that order of magnitude are capable of diffusing rapidly in the human airway mucus through the mucus pores [7,8]. Because of their large surface area in relation to their volume and thereby potential for a large chemical reaction, NPs are believed to exert higher toxicity than larger particles, e.g., fine articles (PM_2.5_) or particulate matter (PM_10_) [9], and have the potential to deteriorate every cell in the human body, as tiny inhaled nanoparticles are translocated into the blood stream and transported around the body [10].

Sick building syndrome (SBS) refers to medical symptoms related to the indoor (work) environment [11,12], particularly the time spent inside a building, but where organ pathology cannot be identified. The concept is rather attributed to a large number of symptoms including headache, dizziness, nausea, mucosal and skin irritation, cough, hoarseness, difficulty in concentration, sensitivity to odors, allergies, and asthma attacks. Questionnaire data from 73 employees showed that irritation of the eyes, a stuffy or runny nose, headache, and drowsiness were the most common SBS symptoms, and often experienced only in connection with the stay in the building [13].

In general, SBS seems to be multifactorial [14,15,16]. Although there is empirical and theoretical support for several psychobiological mechanisms underlying SBS [17], the condition is not completely clarified [18]. Interacting factors include, on the one hand, individual characteristics such as hyper reactivity, sick leave, psychosocial factors, smoking etc. [19,20]. On the other hand, several contributing risk factors have been identified, such as health status, sleep problems, different exposures, and job characteristics [21]. It has been shown that increased stress at work is associated with decreased perceived air quality [22,23]. Additionally, particulate air pollution including NPs may play an important role [24,25,26].

Compared with conventional houses, particularly in new, highly insulated, airtight houses, chemical substances are more likely to accumulate and might induce health symptoms [27]. Dampness, formaldehyde, and alpha-pinene significantly have been associated with SBS symptoms in new buildings [28]. Associations between respiratory function and some types of VOCs have been found even after adjustments for tobacco smoke exposure and other covariates [29]. Fadilah and Jalaudin [30] demonstrated that the prevalence of SBS symptoms (i.e., stuffy or runny nose or sinus) was significantly higher in a new building compared to an old building, despite the fact that the concentration of ultrafine particles was significantly higher in the old building.

Besides direct exposure, the mere perception of pollution may cause health symptoms as a protective mechanism. If the source is recognised based on e.g., its odorous properties and an association of the properties with prior experience is established, the tone of the association (pleasant or unpleasant), will determine whether stress-induced physiological activity in the autonomic nervous system and related brain regions will be lower or higher than normal, respectively [31]. If the recognised source is perceived as unpleasant, it is likely to have a negative impact on health, e.g., evoke annoyance, worry, and disgust as a protective mechanism [32]. Apart from olfaction, the trigeminal sensory system plays a critical role in this context since it, in addition to vaporous substances, is activated by particles. Trigeminal chemoreception generates sensations of pungency and irritation, with the primary function of acting as a sentinel of the airways where it reflexively stops inspiration to prevent inhalation of potentially life-threatening substances [33]. Among other factors that may amplify perception of pollution, increased stress at work is associated with decreased perceived air quality [22,23].

Furthermore, the risk perception (the subjective judgement of whether exposure to an environmental factor poses a health risk) has been shown to be an important factor in the onset of symptoms related to SBS [34]. The worry due to health risk perception may trigger a top-down mechanism involving higher-order neural processing in the brain, which affects emotions, physiology, behaviour and health. Beliefs about a certain chemical/physical exposure being hazardous (irrespective of actually being hazardous or not) and the worry and stress this evokes can contribute to health symptoms [14,15,16]. In a population-based questionnaire study, performed before and after closure of a sinter plant, the participants were asked about health symptoms and risk perception. After the sinter plant had closed, the participants’ perceived health risk and annoyance due to particles and odorous substance, had declined. However, no difference in reported health symptoms was found between the assessments conducted before and after the closure [35]. Similar findings have also been shown by Claeson et al. [36] with people living close to a biofuel facility that emitted odorous substances and by Orru et al. [37] in a population-wide study being exposed to various levels of PM_10_. In both of these studies, the exposure level did not directly affect symptoms; instead, the association was mediated by perceived pollution and health-risk perception. Factors that are important in terms of risk perception include social status and level of control a person considers to have over their own well-being [37]. Nevertheless, there is currently no knowledge on how nanoparticle air pollution risk perception can modify those associations with perceived air quality and SBS.

In our previous analysis, NPs were measured in office buildings in Umeå in northern Sweden as a part of the NanoOffice project [5]. Using data from that project, the aim of this study was to test a path-analytic model that describes the interrelations between NP pollution, perceived air quality, health risk perception, stress, chronic disease and sick building syndrome. According to the model, air pollution leads to perceived pollution, symptoms and disease, perceived pollution leads to health risk perception and symptoms, and health risk perception leads to symptoms and disease. In previous studies investigating complaints from air pollution [35,36,37], an initial model was tested, which included inter-relations between air pollution, perceived pollution, health risk perception, annoyance, and health symptoms. It appeared that relatively low ambient air pollution concentrations did not significantly increase symptoms, yet the perceived exposure influenced health symptoms, and the effect of perceived exposure on disease was mediated by health risk perception [37]. The present study aimed at testing a model for office environment, and particularly for the NP levels, regarding which there is limited knowledge so far. Notably, although the model shows arrows that indicate the most likely direction, the cross-sectional nature of the data does not enable identification of causal effects.

## 2. Materials and Methods

The number concentrations and size distributions of NPs were measured indoors in twelve office buildings in the city of Umeå in 2017 and 2018 (Table 1). The measurements were conducted for a one-week period in each building during the heating and the non-heating season using the SMPS 3938 (SMPS = Scanning Mobility Particle Sizer Spectrometer, TSI Inc., Shoreview, MN, USA). This measurement technique combines electrical mobility sizing with single-particle counting to deliver nanoparticle concentrations in separate size channels. During the measurements, the windows were closed, and no employees were in the rooms due to noise annoyance caused by the measurement device. The office buildings were selected to include new, retrofitted, and old buildings with various ventilation systems and different energy consumption as well as with different distances from a busy street. More details about the NP measurements methodology including measurement placement are given in Orru et al. [5].

Besides measurements of NPs, a questionnaire survey was conducted with the office workers in 2018. The questionnaire included questions about the work environment and conditions, perceived symptoms, risk perception, diagnoses, and stress level. The questionnaire was sent to 469 persons of which 260 responded (55.4%).

In the first step, the measured NP concentrations in the twelve office buildings during the heating season were divided into four quartiles representing low (1), moderate (2), high (3), and very high (4) exposure concentrations (Table 1). Since the questionnaire was responded to during three months of winter (heating season), the latter analyses were based on the NP concentrations measured during that season. The mean exposure concentrations based on the four quartiles were compared with respect to sex, age, and educational level, and tested with two-sample *t*-test.

In the second step, perceived air quality, risk perception, and beliefs among the participants regarding various factors related to the work environment were measured with respect to how concerned they were regarding their impact on their personal health. The ‘perceived air quality’ was based on the question “*What do you think about the air quality at the work place?*” from the Örebro MM 040 Office questionnaire [38] and rated on the five-point scale: “1 = *Very good*; 2 = *Good*; 3 = *Acceptable*; 4 = *Bad*; and 5 = *Very bad*”. The risk perception of nanoparticles was based on the question “*How concerned are you about the influence of the following aspects of your personal health? …very small particles (up to 100 nm) in the indoor air*” based on the KesTeRisk questionnaire [39] and rated on the five-point scale: “1 = *Not at all*; 2 = *A little*; 3 = *Partly*; 4 = *Quite a lot*; and 5 = *Extremely much*”.

**Table 1:** Mean NP concentrations (particles per cm^3^) of two one-week measurement periods in the heating and the non-heating season. The exposure levels are divided into four quartiles based on the concentrations during the heating season.

Building	Mean NP Concentration during Heating Season (Particles per cm^3^)	Mean NP Concentration during Non-Heating Season (Particles per cm^3^)	Exposure Level (Quartiles)
1	3288	869	Very high (4)
2	316	323	Low (1)
3	911	1170	High (3)
4	182	649	Low (1)
5	253	726	Low (1)
6	459	1241	Moderate (2)
7	398	463	Moderate (2)
8	396	566	Moderate (2)
9	575	1139	High (3)
10	1127	879	Very high (4)
11	2211	575	Very high (4)
12	465	598	High (3)

In the third step, long-term stress was quantified based on the 14-item Shirom-Melamed Burnout Measure (SMBM) [40] of the instrument assesses “physical fatigue”, “cognitive weariness“ and “emotional exhaustion” [41]. The Örebro MM 040 Office questionnaire [38] includes a total number of 13 symptoms (e.g., fatigue) experienced during the last three months, rated on the three-point scale: “1 = *Yes, often (every week)*; 2 = *Yes, sometimes*; and 3 = *No, never*”. The symptom was regarded as a SBS symptom if the participant also answered “*Yes*” to the question: “*Do you believe that it is due to your work environment?*”. The measure of chronic diseases was based on the question “*Have you been diagnosed by a physician for the following conditions* … e.g., *high blood pressure*”. The list of physician-based diagnoses was based on the “Västerbotten Environmental Health Study”, in which the list has previously been applied in the similar population [42].

In the fourth and final step, two path analysis models were developed with SPSS Amos 26 (IBM Corp, Armonk, NY, USA). They were based on structural equation modelling in order to test and estimate the relationships between statistical data and qualitative causal assumptions. In this context, the analysis is based on a hypothesis that the three factors ‘Nano-particle concentrations’, ‘Perceived air quality’, and ‘Risk perception of nanoparticles’ influence the health outcomes including ‘Stress symptoms’, ‘Chronic diseases’, and ‘SBS symptoms’. In this confirmatory rather than exploratory modelling, the assumed relations are represented as a model with operationalized constructs that was tested. A value for each construct in the path analysis from each respondent was obtained by ratings. We also used the number of SBS symptoms and chronic diseases as a continuous variable to exploit its full variability. Path coefficients and their *p*-values were calculated in order to estimate the relationships between these constructs. The comparative fit index (CFI), root mean square error of approximation (RMSEA), and *p*-value of close fit (PCLOSE) were used in order to test the goodness of fit between the calculated models and hypothesized models. CFI can take values between 0 and 1, where 1 indicates a perfect fit, and a value close to 0.95 is needed to ensure an acceptable model fit, discussed by Hu and Bentler [43]. RMSEA calculates the model fit using chi-squared tests and the degrees of freedom of the model. A value smaller than 0.06 is needed to ensure a relatively good fit between observed data and a hypothesized model by Hu and Bentler [43]. PCLOSE is a *p*-value for testing the null hypothesis that the RMSEA is no greater than 0.05 [44].

## 3. Results

### 3.1. Description of the Participants

Table 2 gives a general overview of the results from the survey among the employees from different office buildings.

The mean proportion of males and females across buildings was similar. The mean age of participants was slightly below 50 years. The perceived air quality was in general between good “2” and acceptable “3”, being best in building 1 and worst in building 8. There was little concern on NPs; only employees of building 9 were on average partly concerned. The mean stress scores were highest in buildings 8 and 9 and lowest in buildings 11 and 12. On average, each participant had one chronic disease. The prevalence of any SBS symptom varied among buildings. Whilst only 7% of the participants had SBS in building 3, more than 1/3 had it in buildings 8 and 9.

### 3.2. Difference in Degree of Exposure between Demographic Groups

In Table 3, the mean exposure concentration, based on mean value of these quartiles, are presented as different groups with respect to sex, age, and educational level. Two-sample *t*-tests with two-tailed *p*-values were used in order to test whether there were statistically significant differences in the degree of exposure between the groups examined in pairs. No significant difference in exposure between sex and age groups were found, but participants with a doctoral degree were exposed to NPs to a significantly less extent.

### 3.3. Difference in Risk Perception

**Figure 1:**
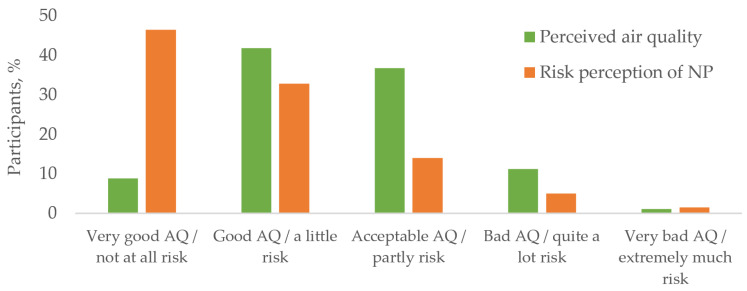
Perceived air quality and risk perception of nanoparticles in the study group.

Figure 1 indicates the perceived air quality and risk perception of nanoparticles. It shows, that in the majority of cases, the air quality in the offices is good or acceptable. Around 1/10 of people viewed the air quality as bad and a few people viewed it as very bad. Regarding risk perception, almost half of participants did not see NP as a health risk for them. Around ⅓ saw this as a small risk and less than 1/10 quite a high health risk.

Table 4 represents the degree of risk perception regarding ‘nanoparticles indoors’ among the participants in the twelve office buildings in different demographic groups. The analyses showed no statistically significant difference between sexes, age groups, and educational levels in the risk perception of nanoparticles indoors.

### 3.4. SBS Symptom Prevalence and Correlations between Symptoms and NP Concentrations

Table 5 presents the prevalence of different SBS symptoms among study participants. The most common SBS symptom was fatigue, of which 29.6% of the participants with any of the SBS symptom reported. It was followed by dry or itching hands and difficulties concentrating (both 11.9%). Nausea, cough, and nose bleeds were the least prevalent and were rarely believed to be due to one’s work environment, but could be classified as an SBS symptom.

The correlations between NP concentration (during the heating season divided into four quartiles) and SBS symptoms are presented as R-values with their significance level (*p*-value). In general, the correlation between measured NP exposure and SBS symptom was low (R = 0.01–0.16). The statistically significant (*p* < 0.05) correlation was seen only for ‘difficulties concentrating’ and appeared for ’feeling heavy headed’. Borderline significant results appeared for ‘nausea/dizziness’ (*p* = 0.06) and ‘irritated eyes’ (*p* = 0.09).

### 3.5. Path Analytic Models Describing the Relationships between Factors

**Figure 2:**
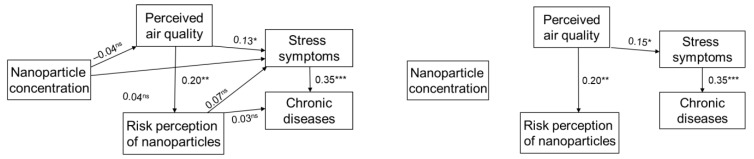
Path analytic model with the relationships between NP concentration, perceived air quality, risk perception of nanoparticles, stress symptoms, and chronic diseases. Presented with both statistically significant and non-significant paths to the left, and with only statistically significant paths to the right. Standardized path coefficients are given under the following conditions: * *p* < 0.05; ** *p* < 0.01; *** *p* < 0.001.

**Figure 3:**
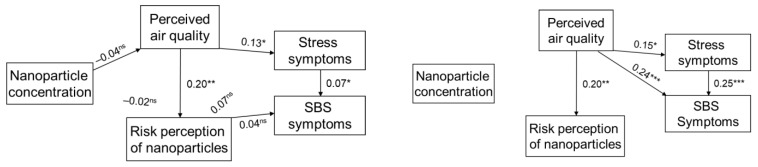
Path analytic model with the relationships between NP concentration, perceived air quality, risk perception of nanoparticles, stress symptoms, and SBS symptoms. Presented with both statistically significant and non-significant paths to the left, and with only statistically significant paths to the right. Standardized path coefficients are given under the following conditions: * *p* < 0.05; ** *p* < 0.01; *** *p* < 0.001.

Following the theorized relations, we tested the model, in which the NP concentration, perceived air quality, and risk perception of nanoparticles are assumed to lead to health outcomes, including stress symptoms, and chronic diseases (Figure 2) and SBS (Figure 3) in the path analyses.

In Figure 2, concerning the stress and chronic disease outcomes, all correlation coefficients and their *p*-values between these factors are presented in the model to the left, whereas only the factors that are statistically significant are presented in the model to the right. The model has a good fit (CFI = 1.00; RMSEA = 0.00; PCLOSE = 0.70). Figure 2 (right panel) also shows the model in which the non-significant paths are excluded. This model did not change substantially from the initial theoretical model and did still indicate a good fit (CFI = 1.00; RMSEA = 0.00; PCLOSE = 0.81). Thus, the model in the right panel of Figure 2 was considered the final model.

The same principle applies to Figure 3, but with the difference that sick building syndrome is included as the health outcome instead of chronic diseases. The model has a good fit (CFI = 0.99; RMSEA = 0.02; PCLOSE = 0.42). Figure 3 (right panel) also shows the model in which the non-significant paths are excluded. This model did not change substantially from the initial, theoretical model and did still indicate a good fit (CFI = 1.00; RMSEA = 0.00; PCLOSE = 0.63). Thus, the model in Figure 2 was considered the final model.

## 4. Discussion

### 4.1. Nanoparticle Concentration and SBS Symptoms

The aim of this study was to test a model of interrelations between NP pollution, perceived air quality, health risk perception, stress, chronic disease, and sick building syndrome. The relationships between the NP concentration and the different factors related to chronic diseases and SBS, presented in the path analytic models (Figure 2 and Figure 3), exhibit varying degrees of correlation. Results from the path analysis showed that the level of NP exposure did not significantly influence the perceived pollution and health risk perception, stress, SBS, or chronic diseases. Perceived exposure was found to influence the health risk perception. Perceived exposure was found to influence stress, which in turn influenced both SBS and chronic disease. This is similar to earlier studies applying path models, where no effect of direct exposures has been seen [36,37]. This and earlier studies have shown that perceived pollution plays an important role in understanding and predicting health symptoms and/or chronic diseases [36,37,45]. When we tested two different models, both the model applying SBS as the outcome as well as the model applying the chronic diseases had a good fit. However, compared to chronic diseases, SBS was more strongly linked with perceived air quality. This might be due to the fact that SBS symptoms appear earlier and more easily than chronic diseases. These results are well aligned with earlier findings suggesting that individuals with higher sensitivity to air quality tend to respond more to exposure to poor air quality that may pose an increased risk of developing SBS symptoms [46]. The non-significant effect of air pollution on the other factors in the model may also be attributed to the fact that NPs are practically invisible and do not smell.

Caution should be taken regarding the direction of effects in the proposed model. Path analysis does not test the cause–effect direction between factors per se. For example, it cannot be excluded that perceived pollution and health risk perception or SBS and health risk perception mutually affect each other. Moreover, we cannot exclude that other effects, besides the air pollution itself, e.g., the psycho-social climate in the office, may amplify the perceived exposure and risk perception. For example, increased stress at work has been associated with decreased perceived air quality [38,39]. In a study in Asia, better mental health was related to lower levels of perceived air pollution [47]. In addition, in the Netherlands, parental worry was shown to be positively related to perceived risks associated with air pollution [48]. In an previous study, preceived air quality was found to be associated with perceived health risks [49]. In the current study, we could also see that perceived exposure is more closely linked to health-related factors than the NP exposure itself. However, the directions of cause–effect are not clarified, and high stress levels may contribute to higher risk perception, but the reverse relationship is also possible.

Furthermore, positive aspects of the perceived working environmet, e.g., nice ambiance may overdraw negative aspects of pollution, particulalry if it cannot be identified with human senses, as is the case with NPs. For example, the air quality was perceived to be the best in Building 1, despite the fact that the highest mean NP concentration during the heating season was measured in that building. The new Building 1 with modern facilities and ventilation may foster beliefs of a healthy working environment. Earlier studies, e.g., by Acar and Göç [50], argue that risk perception is affected by owners’ psychological state, which might be modified by building characteristics. For instance the prevalence of SBS has been shown to be lower in green buildings [51]. The results are likely to have been affected by the rather low levels of air pollution, which are also reflected by the low ratings of perceived air pollution (mean scores in buildings range: 1.6–2.9 on a scale ranging from no exposure (1) to high exposure (5)), health risk perception (1.4–2.8), and stress (0.1–0.7 on a scale of 0 to 3 stress symptoms).

When comparing males and females, or different age groups, no significant difference was found in their exposure. As both men and women work in similar offices, the gender neutrality appears to be expectable. If we see different work places or different areas in cities, the gender differences in air pollution exposure have been reported in a small number of studies [52]. However, significantly lower NP exposures appeared among individuals with a doctoral degree compared to lower educational groups. This indicates that the group that had a doctoral degree generally worked in environments with relatively lower NP concentrations. Although several studies have shown higher exposure among groups with lower education and sociodemographic level [53,54,55], to our knowledge, there have not been studies comparing different groups among highly educated persons. There were no differences in risk perception between different groups of sex, age, or educational levels.

The findings suggest that perceived pollution and health risk perception play significant roles in understanding and predicting indoor environmentally induced symptoms and chronic diseases. Perceived air pollution may have resulted in negative affect, stress-induced physiological activity, and thereby health symptoms. Health risk perception involves top-down defence mechanisms that may evoke symptoms and contribute to diseases. If the individual believes that the exposure is hazardous, this will result in symptoms and disease that may guide an individual to avoid the exposure. The impact of health risk perception on health has been demonstrated in several well-controlled experimental studies (e.g., [56,57]).

As for potential direct health effects of NP exposure, among the studied 13 SBS symptoms, “feeling heavy headed” and “difficulties concentrating” are the two symptoms that showed statistically significant associations with the NP concentration. The association between “nausea/dizziness” and NP concentration was borderline significant. What is notable in this context is that these three symptoms that show the strongest association with NP concentration are all directly linked to the central nervous system. Since NPs are capable of crossing the blood–brain barrier as well as travelling via the olfactory nerves from the nose to the brain, they are therefore also potentially hazardous to the CNS [58,59,60,61]. Neurotoxic effects caused by NPs can occur via several mechanisms including oxidative stress, autophagy, lysosome dysfunction, and through the activation of certain signaling pathways [62]. Although the above-mentioned associations are statistically significant, the correlations are relatively weak. Furthermore, these associations did not remain significant when the perception of air quality and risk perception, which are more closely associated with SBS, were taken into account in the path analysis.

The relatively low levels of perceived risk from NPs identified in this study may be time- and context-specific as there is increasing concern about nanosafety for public and occupational health [63], particularly in the context of increasing use of smart materials and nanotechnology [64]. However, there is scarce information on the number of workers exposed to nanoparticles and nanomaterials in the workplace, including offices, or the effects of such exposure on human health.

### 4.2. Strengths and Limitations of This Study

A strength of this study is that it includes twelve different buildings with a wide variety of mean measured NP concentrations. A recent WHO global air quality guideline analysis has pointed out the importance of quantifying the concentration of particles < 100 nm and utilized this in epidemiological studies, as such studies are currently scarce, and the latter applies to air quality management [65]. A total number of 260 office workers (with response rate of 55%) who responded to the survey may be considered as a sufficient sample for analyses. A total number of 13 SBS-related symptoms were addressed in the survey, which provides a good basis for identifying possible associations with SBS.

According to our path analysis, the perception of air quality and risk perception have a larger role in SBS compared to “real” (chemical) exposures. However, with the current study methodology, the relatively small sample size and low levels of NP concentrations, we cannot fully exclude the physiologically mediated impacts, through, e.g., airway irritation, which may exist regardless of the effect of perception. Further studies may explore the potential health effects of higher levels of NP concentrations, including with different chemical compositions. In this study, the chemical compositions of the NPs have not been analyzed. The chemical compositions of NP can be expected to vary greatly between the twelve office buildings depending on the infiltration of outdoor air, proximity to busy roads, indoor activities, and building materials. Different chemical compositions can be assumed to have different effects on SBS-related symptoms [46,66,67], but these effects could unfortunately not be identified as only the concentrations have been measured. Additionally, other pollutants, such as VOCs, may play an important role in triggering SBS. Furthermore, the effect of the psycho-social environment indoors needs further scrutiny as a potential driver of perceived risks from air pollution mediating health effects.

## 5. Conclusions

The path analyses suggest that perceived air quality plays a very important role in understanding and predicting stress, SBS, and diseases. In the studied office buildings, the total NP concentrations were correlated with two SBS symptoms, ‘feeling heavy headed’ and ‘difficulties concentrating’, both of which are directly related to the central nervous system. However, these associations between NP and SBS did not remain significant when the perception of air quality and risk perception were included in the path analysis. This confirms the strong health impacts of health risk perception, which may be related to, e.g., beliefs about a certain chemical/physical exposure being hazardous or a poor psycho-social climate in the building. According to our study, health risks may increase if NPs are recognized and perceived as unhealthy by employees. Cautiously informing people about the health effects of NP exposure is necessary in order to avoid triggering additional health consequences due to health risk perception. Future larger-scale studies may explore whether physiologically mediated impacts, e.g., through airway irritation, exist regardless of the effect of perception. Moreover, the health effects of higher levels of NP concentrations including with different chemical compositions or in co-existence with VOCs may be tested.

## Figures and Tables

**Table 2:** A general overview of the results from the written surveys with the employees in the twelve buildings.

Building	Males (%)	Age (Mean)	Perceived Air Quality (Mean) ^1^	Risk Perception of NP (Mean) ^2^	Stress Score (Mean) ^3^	Number of Chronic Diseases (Mean)	Number of SBS Symptom (Mean)
1	50	51	1.6	1.6	0.6	1.2	0.2
2	50	48	2.4	1.7	0.3	1.3	0.4
3	53	47	2.3	1.7	0.5	0.7	0.1
4	35	39	2.6	1.5	0.6	0.4	0.4
5	63	50	2.5	1.9	0.4	1.0	0.8
6	80	48	2.8	1.4	0.3	1.1	0.2
7	38	45	2.2	1.7	0.5	0.7	0.1
8	23	53	2.9	2.1	0.7	1.3	0.8
9	58	38	2.7	2.8	0.7	0.6	0.5
10	47	47	2.5	1.7	0.3	1.1	0.4
11	8	46	2.8	2.0	0.2	1.5	0.1
12	55	48	2.4	1.6	0.1	1.0	0.1
Mean	47	47	2.5	1.8	0.4	1.0	0.3

^1^ Perceived air quality is graded according to the following: “1 = very good, 2 = good, 3 = acceptable, 4 = bad, and 5 = very bad”. ^2^ Risk perception for nanoparticles is graded according to how concerned they were according to the following: “1 = not at all, 2 = a little, 3 = partly, 4 = quite a lot, and 5 = extremely much”. ^3^ The stress score is based on the mean sum of the presence of (1) or absence of (0) “physical fatigue”, “cognitive weariness” and “emotional exhaustion”, which gives a sum from 0 to 3.

**Table 3:** Mean NP exposure concentrations for different demographic groups. Paired *t*-tests with *p*-values indicate the differences between the mean values where a *p*-value < 0.05 indicate a statistically significant difference between the groups.

Groups (1 vs. 2)	Mean Value for Group 1	Mean Value for Group 2	*t* Stat	*p*-Value (Two-Tailed)
Males vs. females	2.43	2.55	−0.85	0.40
Ages 18−39 vs. 40−49	2.53	2.58	−0.28	0.78
Ages 18−39 vs. 50+	2.53	2.42	0.66	0.51
Ages 40−49 vs. 50+	2.58	2.42	1.02	0.31
Secondary school vs. College/University	2.83	2.58	1.08	0.28
Secondary school vs. Doctoral degree	2.83	2.29	2.38	0.02
College/University vs. Doctoral degree	2.58	2.29	1.98	0.05

**Table 4:** Mean values for risk perceptions regarding “nanoparticles indoors” divided into groups in pairs. *t*-tests with *p*-values indicate the differences between the mean values where a *p*-value < 0.05 indicate a statistically significant difference between the groups.

Groups (1 vs. 2)	Mean Value for Group 1	Mean Value for Group 2	*t* Stat	*p*-Value (Two-Tailed)
Males vs. females	1.80	1.83	−0.21	0.83
Ages 18−39 vs. 40−49	1.90	1.77	0.78	0.44
Ages 18−39 vs. 50+	1.90	1.81	0.62	0.54
Ages 40−49 vs. 50+	1.77	1.81	−0.25	0.80
Secondary school vs. College/University	1.83	1.87	−0.22	0.83
Secondary school vs. Doctoral degree	1.83	1.78	0.24	0.81
College/University vs. Doctoral degree	1.87	1.78	0.69	0.49

**Table 5:** Prevalence of SBS symptoms and correlations between symptoms and NP concentration as well as significance levels.

SBS Symptoms	Prevalence (%)	Correlation Coefficient (R-Value)	Significance Level (*p*-Value)
Fatigue	29.6	0.08	0.24
Feeling heavy headed	10.7	0.13	0.05
Headache	10.7	0.03	0.60
Nausea/dizziness	3.3	0.13	0.06
Difficulties concentrating	11.9	0.16	0.02
Irritated eyes	10.4	0.11	0.09
Irritated nose	10.7	0.04	0.55
Nose bleeds	0.7	0.04	0.59
Hoarseness and dry throat	4.1	0.07	0.30
Cough	2.6	0.01	0.92
Dry or flushed facial skin	8.5	0.07	0.31
Scaling of scalp or ears	4.8	0.06	0.41
Dry or itching hands	11.9	0.08	0.24

## Data Availability

The data presented in this study are available on request from the corresponding author.
